# Mitochondrial Dysfunction in *Pten* Haplo-Insufficient Mice with Social Deficits and Repetitive Behavior: Interplay between Pten and p53

**DOI:** 10.1371/journal.pone.0042504

**Published:** 2012-08-10

**Authors:** Eleonora Napoli, Catherine Ross-Inta, Sarah Wong, Connie Hung, Yasuko Fujisawa, Danielle Sakaguchi, James Angelastro, Alicja Omanska-Klusek, Robert Schoenfeld, Cecilia Giulivi

**Affiliations:** 1 Department of Molecular Biosciences, School of Veterinary Medicine, University of California Davis, Davis, California, United States of America; 2 Medical Investigations of Neurodevelopmental Disorders Institute, School of Medicine, University of California Davis, Davis, California, United States of America; University of Texas Health Science Center at San Antonio, United States of America

## Abstract

Etiology of aberrant social behavior consistently points to a strong polygenetic component involved in fundamental developmental pathways, with the potential of being enhanced by defects in bioenergetics. To this end, the occurrence of social deficits and mitochondrial outcomes were evaluated in conditional *Pten* (Phosphatase and tensin homolog) haplo-insufficient mice, in which only one allele was selectively knocked-out in neural tissues. *Pten* mutations have been linked to Alzheimer's disease and syndromic autism spectrum disorders, among others. By 4–6 weeks of age, Pten insufficiency resulted in the increase of several mitochondrial Complex activities (II–III, IV and V) not accompanied by increases in mitochondrial mass, consistent with an activation of the PI3K/Akt pathway, of which Pten is a negative modulator. At 8–13 weeks of age, *Pten* haplo-insufficient mice did not show significant behavioral abnormalities or changes in mitochondrial outcomes, but by 20–29 weeks, they displayed aberrant social behavior (social avoidance, failure to recognize familiar mouse, and repetitive self-grooming), macrocephaly, increased oxidative stress, decreased cytochrome *c* oxidase (CCO) activity (50%) and increased mtDNA deletions in cerebellum and hippocampus. Mitochondrial dysfunction was the result of a downregulation of p53-signaling pathway evaluated by lower protein expression of p21 (65% of controls) and the CCO chaperone SCO2 (47% of controls), two p53-downstream targets. This mechanism was confirmed in Pten-deficient striatal neurons and, HCT 116 cells with different p53 gene dosage. These results suggest a unique pathogenic mechanism of the Pten-p53 axis in mice with aberrant social behavior: loss of Pten (via p53) impairs mitochondrial function elicited by an early defective assembly of CCO and later enhanced by the accumulation of mtDNA deletions. Consistent with our results, (i) SCO2 deficiency and/or CCO activity defects have been reported in patients with learning disabilities including autism and (ii) mutated proteins in ASD have been found associated with p53-signaling pathways.

## Introduction

Phosphatase and tensin homolog on chromosome ten (*Pten*) is a tumor suppressor gene mutated in many human cancers [1]. While loss of both alleles seems relevant for tumorigenesis, clinical expressions of inherited *Pten* mutations had been linked to benign hamartomas, macrocephaly, seizures, ataxia, mental retardation, autism and, more recently, to Alzheimer's disease [1]–[10]. *Pten* mutations have been reported in cases of autism, particularly in a subset of patients with macrocephaly, suggesting that mutations in this gene might be one of the genetic risk factors for human ASDs [9]–[13]. Kwon et al [14] showed that conditional *Pten* null mice resulted in impaired social interaction and learning, no preference for social novelty, limited nest-forming activity, as well as abnormal anxiety levels [14]. These mice showed activation of the Akt/mTOR/S6k pathway and inactivation of GSK3*β* [15] suggesting that abnormal activation of the PI3K/Akt pathway in specific neuronal populations could underlie the observed macrocephaly and behavioral abnormalities. In another study, the *GFAP-Cre* transgenic line used to drive Cre activity in *Pten* cKO mice induced conditional deletion of *Pten* in astrocytes as well as in some neuronal populations (hippocampal, cerebellar granule and pyramidal neurons [16]). Neurons from these >10-weeks old mice exhibited increased mitochondrial size (megamitochondria) along with defects in synaptic structures and myelination [17]; however, no data on mitochondrial function or activities is currently available for this model. Of interest, it has been reported that overexpression of Pten had a profound effect in intermediary metabolism of mice, increasing their energy expenditure and decreasing fat deposits [18], suggesting a link between Pten and energy metabolism.

The present study was designed to expand earlier work performed with the conditional *Pten* (phosphatase and tensin homolog) null mouse by studying mitochondrial function in 3 brain regions, namely cortex, hippocampus and cerebellum. To this end, social and repetitive behavior was tested in heterozygous *Pten* ^+/loxP^ (HET) and heterozygous *Nse-*cre^+/−^; *Pten* ^+/loxP^ (HET-CRE) mice aged 4–29 weeks (**Table 1**), along with the evaluation of mitochondrial enzymatic activities, mtDNA copy number and mtDNA deletions, in cerebellum, hippocampus and cortex. Considering that a subset of individuals with ASD who presented macrocephaly had only one mutated *Pten* allele [9], [19], [20], not a complete knock-out as used in Kwon *et al.* [14], we used conditional *Pten* knock-out mice in which only one allele of *Pten* was selectively knocked-out in neurons to better resemble the genotype and phenotype of these patients without increasing significantly the risk or incidence of cancer which may obscure the outcomes.

**Table 1:** Characteristics of the mouse groups and tests performed.

Age range (weeks)	Tests performed	HET-CRE				HET				*p*-value
		*n*	F	M	Age (wk)	*n*	F	M	Age (wk)	
4–6	BMB, H,	7	4	3	4.6±0.1	8	4	4	4.9±0.2	0.178
8–13	BMB, H, B, BW	7	4	3	10.7±0.5	11	5	6	10.8±0.4	0.947
20–29	BMB, B, BW	6	3	3	24±3	10	5	5	24.5±0.8	0.759

The age range represents the 95% Confidence interval age limits in weeks and the age of the group in weeks was expressed as mean ± SEM. Abbreviations: BMB, molecular tests in biochemistry and molecular biology; H, histology; BW, brain weight; B, behavior; F, female, M, male. The *p*-values were obtained by comparing the age of HET-CRE to that of HET.

Our study is the first one at providing a thorough and comprehensive analysis of mitochondrial activities during Pten haplo-insufficiency and a mechanism based on a negative feedback loop between two tumor suppressors, Pten and p53, that results in altered bioenergetics in mice with social deficits.

## Results

### *Pten* haplo-insufficiency in cerebellum and hippocampus of mice

Conditional *Pten* knock-out mice were obtained by flanking exon 5, which encodes the phosphatase domain of *Pten*, between LoxP sequences [21]. To generate a Cre-mediated exon 5 deletion of *Pten*, we crossed *Pten^loxP/loxP^* animals with the Nse-Cre^+/−^ transgenic mice aimed for neural tissue-specific deletion. The mice utilized in this study were considered controls (i.e., named HET for heterozygous *Pten* ^+/loxP^) or Pten-haploinsufficent (i.e. named HET-CRE for heterozygous *Nse-*cre^+/−^; *Pten* ^+/loxP^), aged 4 to 29 weeks.

**Figure 1:**
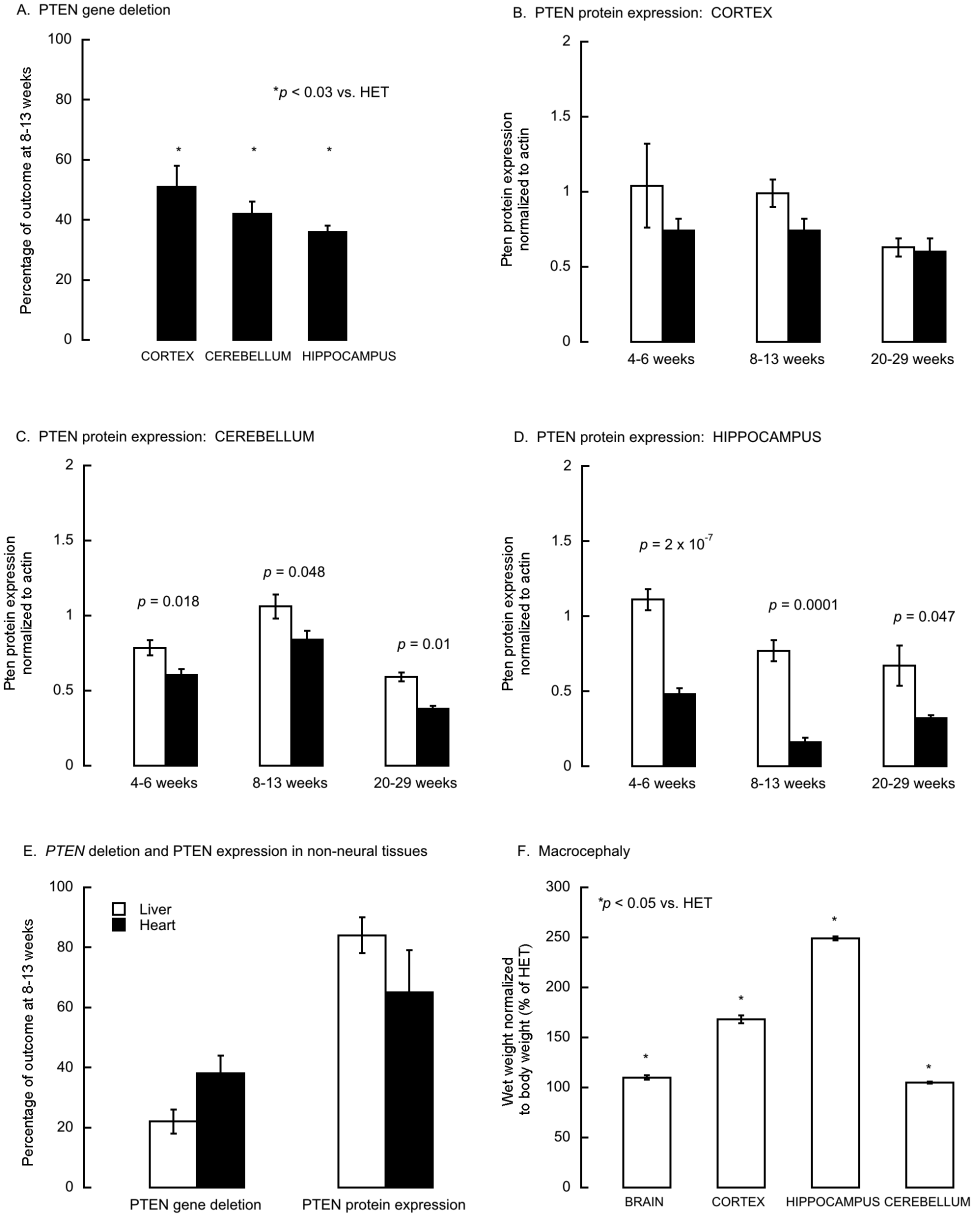
Changes of Cre-mediated expression of Pten protein and gene with age in brain from *Pten* haplo-insufficient mice. HET and HET-CRE groups were sex- and age matched and at each age and each group had 7 to 11 animals (See **Table 1**). All values were expressed as mean ± SEM. **Panels A–D**: HET, white bars; HET-CRE, black bars. **Panel A**: *Pten gene deletion in various brain regions from HET-CRE mice aged 8–13 weeks*. Pten gene deletion was calculated as the ratio of the truncated band over that of the normal allele for Pten exon 5 determined as described in the **Methods S1**. **Panels B–D:** *Pten protein expression in brain regions from HET and HET-CRE mice aged 4–6, 8–13, and 20–29 weeks*. The densitometry of the Pten band obtained by Western blots was normalized to that of actin for each brain region. The *p-*values were obtained by using Student's *t*-test. **Panel E:** *Pten gene deletion and Pten protein expression in liver and heart from HET and HET-CRE mice aged 8–13 weeks*. *Pten* gene deletion was obtained as described under **1A** legend. Pten protein expression was calculated as described under **Panel B–D** legend but expressed as percentage of age-matched HET values. **Panel F**: *Assessment of macrocephaly in Pten haplo-insufficient mice*. Mean values for HET mice at 20–29 weeks were 322±15 mg brain wet weight; body weight 24±3 g; 1.7±0.2 mg cerebellum/g body weight; 1.4±0.4 mg cortex/g body weight; 0.66±0.06 mg hippocampus/g body weight. * All values were significantly different from HET with *P*<0.05.

Immunohistochemistry performed in brain slices with antibodies to Cre-recombinase showed that the deletion of *Pten* was mainly restricted to cerebellum and hippocampus (not shown) as reported by others [14], [22]. Cre immunostaining was minimal at early postnatal days, increasing from 8–13 weeks (not shown) coincidentally with the occurrence of *Pten* gene deletion in these brain regions (**Figure 1A** **, Figure S1**). The efficiency of the Cre-mediated deletion of *Pten* in HET-CRE mice aged 8–13 weeks was 43±4% for neural tissues when compared to HET mice (near haploid; **Figure 1A**). Pten protein expression was not statistically different between HET and HET-CRE mice aged 4–6 weeks in cortex (**Figure 1B**), whereas in cerebellum and hippocampus the decrease was significant in HET-CRE (69% of HET, *p*<0.05 and 44% of HET, *p*<0.00005 respectively; **Figure 1C, D**). At 8–13 weeks, Pten protein expression in HET-CRE cerebellum and hippocampus was (average of these two regions) 50±1% of HET values, decrease still sustained at 20–29 weeks (59±1% vs. 8–13 weeks, *p* = 0.356). No significant decrease in Pten levels were observed at any age (4–29 weeks old) in cortex from HET-CRE mice. This result could be explained by the different spatial and temporal expression of Cre in various brain regions [23]–[25]; alternatively, the apparent lack of Pten knockdown in cortex could result from a compensatory response from glia, masking the decrease in neurons. Consistent with this hypothesis, the levels of glial fibrillary acidic protein (determined by western blots normalized to actin) were higher in HET-CRE cortex than in HET mice aged 20–29 weeks (0.50±0.09 vs. 0.13±0.05; *p* = 0.025). As expected, in non-neural tissues (liver and heart), Pten protein expression in HET-CRE (expressed as a percentage of HET values) was not significantly different from HET (**Figure 1E**).

### Development of macrocephaly in Pten-haploinsufficient mice

Brain hypertrophy and overgrowth of cortex and hippocampus were observed in haplo-insufficient *Pten* mice consistent with the macrocephaly reported in *Pten* null mouse model [14] and that observed in children with autism who harbor *Pten* mutations [9], [10], [26]. Macrocephaly and the occurrence of aberrant social behavior was observed in a few mice at 8–13 weeks of age (only 2 of 7 mice presented macrocephaly and 1 of 7 presented abnormal behavior; *n* = 7/group; *p* = 0.433) becoming significantly different at >20 weeks of age. At 20–29 weeks, brain weight (normalized to body weight) was significantly higher in HET-CRE than HET (10%; *p*<0.05; **Figure 1F**). The enlargement of cortex and hippocampus in HET-CRE was respectively 1.7- and 2.5-fold of controls (*p*<0.05; **Figure 1F**), whereas the change in cerebellum was more modest (5%; *p*<0.05; **Figure 1F**). The apparent discrepancy between cortex and hippocampus overgrowth compared to that in cerebellum could be explained by considering brain-region specific outcomes (i.e., tissue-specific distribution of Cre [15] and/or contribution of gliosis). In this regard, gliosis might play a more relevant role in cortex, whereas macrocephaly with neuronal hypertrophy [14]–[16], enlarged caliber of neuronal projections and increased dendritic spine density [14], [17] –as observed in *Pten* conditional KO mice- would in cerebellum and hippocampus given the relatively higher glia-to neuron ratio in cortex than in the other two brain regions (3.7, 0.4 and 0.2 in cortex, hippocampus and cerebellum, respectively [27], [28]). In addition, a reciprocal correlation between cortex frontal lobe and cerebellum sizes has been observed in children with autism [29] indicating interactions between brain regions. The observations performed with our model (one-allele) were consistent with those from conditional *Pten null* mice in which the development of macrocephaly and aberrant social behavior seemed to reflect a gene-dose effect (one- vs. two-allele deletion) rather than an age-dependent onset for the development of abnormal behavior. In this regard, appearance of macrocephaly (7% increase in brain weight) and aberrant social behavior in the conditional *Pten* null model was observed by 1–1.5 months becoming statistically significant at 2 to 3 months of age (17% increase; [14], [15]).

### Social behavior and self-grooming activity in HET-CRE mice

Sociability (the tendency to spend time with another conspecific), preference for social novelty, and the ability to discriminate and choose between familiar and new conspecifics were tested in Het and HET-CRE mice [30], [31].

During the socialization trial, the test mouse is placed in the center compartment of a three-chambered test box, and given a choice between spending time in the side containing an unfamiliar (stranger) conspecific mouse, or spending time in the third of the box occupied by a control object (an empty cup), to measure exploration of something that has no social valence (non-social side). The stranger mouse is contained within a small wire cage (identical to the one occupied by the cup in the non-social side of the box), to allow exposure to visual, auditory, olfactory, and some tactile stimuli, while preventing aggressive or sexual interactions. Measures taken during the trial include number of entries and time spent in each side of the box, and time spent sniffing each wire cage.

Analysis of the scores obtained during the socialization interaction trial showed that, while the number of entries (frequency) in the social side of the chamber were the same in the two groups of mice, HET mice spent more time (1.3-fold; *p* = 0.014) with the social target than HET-CRE mice (social side time; **Table 2**). Two other outcomes were also recorded during the socialization trial: sniff time and sniff frequency. Sniff frequency was defined as the number of times the mouse sniffed the inanimate target and/or the social target. Sniff time was considered as the cumulative time that the test mouse spent sniffing the inanimate target (nonsocial) and/or the social target (social). While no differences were recorded for the sniff frequencies between the two groups, HET mice spent 1.3-fold (*p* = 0.05) more time than HET-CRE mice sniffing the social target (**Table 2**). The social avoidance score in HET mice, defined as the difference between social side and non-social side times, was 3.7-fold of HET-CRE mice (*p* = 0.006). This score underlines the finding that HET-CRE mice exhibit less preference for socialization than HET mice.

**Table 2:** Behavioral tests performed on HET and HET-CRE mice aged 20–29 weeks.

Outcome	HET	HET-CRE	-fold	*p-*value*
SOCIALIZATION TRIAL				
†Social side time	0.58±0.04	0.45±0.04	0.78	0.014
‡Social side frequency	0.41±0.03	0.46±0.03		
Social/(Social+Nonsocial) sniff time	0.60±0.04	0.47±0.06	0.78	0.050
Social/(Social+Nonsocial) sniff frequency	0.56±0.03	0.53±0.08		
Social avoidance/Total side time	0.33±0.06	0.09±0.06	0.27	0.006
NOVELTY TRIAL				
†Novel side time	0.54±0.02	0.45±0.04	0.83	0.041
‡Novel side frequency	0.41±0.01	0.39±0.02		
Novel/(Novel+Familiar) sniff time	0.61±0.01	0.36±0.09	0.59	0.050
Novel/(Novel+Familiar) sniff frequency	0.52±0.02	0.48±0.05		
SELF-GROOMING TEST	32±3	61±3	1.91	0.03

HET and HET-CRE values were expressed as mean ± SEM. The fold increase represents the ratio between HET-CRE and HET means.

* *p*-value calculated using the one-tailed *t*-test between two independent means. For all comparisons, significance was set at *p*≤0.050.

† Normalized by total trial time.

‡ Normalized by total trial frequency.

The novelty preference trial presented the test mouse with a familiar mouse versus a novel one (in place of the cup used during the socialization trial; **Table 2**). Once again, while no difference was observed between the two groups in the number of entries to the novel target, HET-CRE mice had lower novel to total side time ratio (83% of HET; *p* = 0.04; **Table 2**), confirming that HET-CRE mice did not show a preference for social novelty, in agreement with the findings on *Nse*-cre *Pten* ^loxP/loxP^ [14]. During the novelty preference trial, the sniff frequency, defined as the number of times the test mouse sniffed the novel mouse normalized by the total number of times the mouse sniffed both familiar and novel targets, was not significantly different between the two groups. However, the time spent by the test mouse sniffing the novel mouse normalized by the total sniff time was significantly lower in HET-CRE mice (60% of HET; *p* = 0.05) than HET mice (**Table 2**).

The results of the socialization and novelty trials suggested that HET-CRE mice showed a social avoidance preference (more time spent with inanimate object than with mouse and less time at interacting with a novel mouse, respectively) with a failure to recognize the familiar mouse (more time spent with familiar than novel mouse).

A separate behavioral test was used to evaluate repetitive behavior as judged by the time spent by the test mouse in self-grooming activities. In terms of repetitive behavior, HET-CRE mice showed twice the frequency of self-grooming than HET mice (defined as the cumulative time in seconds spent self-grooming scored over a 15-min session; **Table 2**; *p* = 0.03). These results suggested the occurrence of repetitive behavior (or insistence of sameness) in 20–29 weeks old HET-CRE mice.

Although only one HET-CRE mouse (out of 7) started to show aberrant social behavior by 8–13 weeks, none of the behavioral tests were significantly different in this age group.

### Mitochondrial outcomes in hippocampus cerebellum and cortex from asymptomatic, 4–6 and 8–13 weeks *Pten* haplo-insufficient mice

Several mitochondrial outcomes were tested in purified, mitochondria isolated from cortex, cerebellum and hippocampus from 4–6 and 8–13 weeks old *Pten* haplo-insufficient mice.

**Figure 2:**
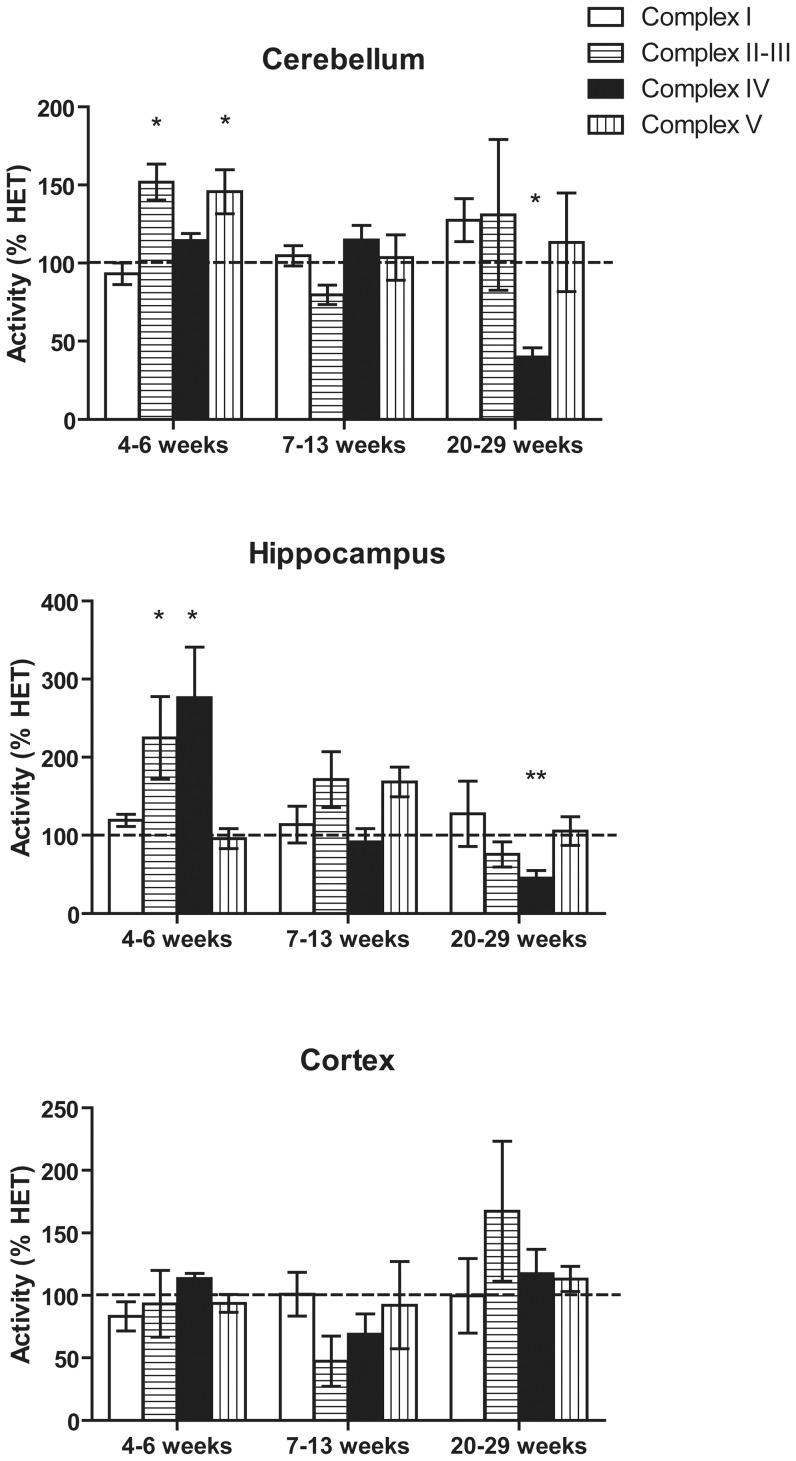
Changes in ETC activities in cerebellum, hippocampus and cortex in HET and HET-CRE mice. Enzymatic activities were evaluated as described in the Materials and Methods section and originally expressed as nmol×(min×mg protein)^−1^ and normalized to citrate synthase activity for each brain region and age. Complex I activity was evaluated by measuring either NFR (cerebellum and hippocampus) or NQR (cortex) activities. Data were expressed as percentage of HET values (mean ± SEM) and analyzed by unpaired *t* test. * *p*<0.05 compared with HET.

In HET-CRE mice 4–6 weeks old, higher Complex II–III (SCCR = 225% of HET values; *p* = 0.045) and Complex IV (CCO = 277% of HET values; *p* = 0.03) activities (hippocampus) and increased Complex II–III (152% of HET; *p* = 0.02) and ATPase (146% of HET; *p* = 0.04) activities (cerebellum) were not accompanied by increased citrate synthase (CS, considered a marker of mitochondrial mass [32]; average of two brain regions, 4–6 weeks = 96±10% of HET) consistent with a lack of mitochondrial biogenesis (**Figure 2**). No significant difference was found in any of the activities tested in the 8–13 weeks old mice in cerebellum or hippocampus. These results were consistent with the findings obtained with the behavioral tests, which did not show any significant difference between HET and HET-CRE at this age group.

No differences in ETC activities were also observed in cortex, at any age (**Figure 2**), consistent with the lack of Pten expression change (**Figure 1B**).

A higher mtDNA copy number (1.45-fold of HET; *p* = 6×10^−6^) was noted in cerebellum from 8–13 weeks old HET-CRE mice, once again not accompanied by increases in CS activity, suggesting a general response to oxidative stress in response to a decreased energy production [33]–[35].

### Mitochondrial outcomes in hippocampus, cerebellum and cortex from symptomatic, 20–29 weeks *Pten* haplo-insufficient mice

Compared to age-matched HET mice, significant decreases in cytochrome *c* oxidase activity (CCO) were observed in HET-CRE cerebellum (59%; *p*<0.05; **Table 3**, **Figure 2**) and hippocampus (35%; *p*<0.05; **Table 3**, **Figure 2**) at 20–29 weeks. The content of nitrated Tyr in ATPase β-subunit, a sensitive marker of nitrative stress [36], in HET-CRE cerebellum was 1.9-fold of HET values (*p* = 0.01; **Figure S2**) suggesting an increased nitrative/oxidative stress. Consistent with this hypothesis, significant mtDNA deletions -at the segments encoding for ND4, COX3 and CYTB- (**Table 3**) and higher MnSOD protein expression (2.5-fold of HET; *p*<0.05 and 1.4-fold of HET; *p* = 0.05) were observed in cerebellum and hippocampus of HET-CRE mice compared to HET (not shown). In contrast with cerebellum and hippocampus, no significant differences were observed in cortex from HET and HET-CRE mice.

**Table 3:** p53 and p53-downstream targets, CCO activity, mtDNA copy number and deletions in *Pten* haplo-insufficient mice aged 20–29 weeks.

Outcome*^a^*	Cerebellum	Hippocampus	Cortex
**mtDNA copy number**	86±8	89±6	91±9
**mtDNA deletions** (a)	15±4*	31±4**	9±1
**Protein expression** (b)			
Pten	65±7*	47±6*	85±20
p53	42±12*	83±3	103±1
p21	54±4**	61±9*	-
SCO2	45±7*	57±12**	-
**Activity**			
CCO/CS	40±6*	45±10**	115±2

All outcomes were expressed as percentages of HET values. The values for the outcomes from HET mice were the following: Hippocampus: mtDNA copy number 1214±178; mtDNA gene ratios for ND4, CYTB and COX3 = 0.86±0.05, 1.22±0.02 and 1.65±0.3, respectively; CCO 235±26 and CS 144±14 nmol×(min×mg protein)^−1^. Cortex: mtDNA copy number 1563±177; mtDNA gene ratios for ND4, CYTB and COX3 = 0.92±0.06, 1.12±0.01 and 1.5±0.2, respectively; CCO 245±19 and CS 170±9 nmol×(min×mg protein)^−1^. Cerebellum: mtDNA copy number 2600±17; mitochondrial gene ratios for ND4, CYTB and COX3 = 0.83±0.06, 1.02±0.002 and 1.1±0.1, respectively; CCO 60±6 and CS 150±4 nmol×(min×mg protein)^−1^.

(a) Percentage of mtDNA deletions were calculated as follows for each of the three tissues: 100−(100×average of mitochondrial gene ratio HET-CRE/HET).

(b) Protein expression was performed by western blot and by normalizing the intensity of the band of the loading control (actin) and expressed as percentage of control values.

* *p*<0.05;

** *p*<0.01;

*** *p*<0.001. Representative western blot images and densitometry results are shown in **Figures S3 and S4**.

### *Pten* haplo-insufficiency, p53 levels and cytochrome c oxidase activity in 20–29 weeks old mice

Given the genetic interaction between *Pten* and p53 [37]–[43], and considering that p53 is an upstream factor for the assembly of CCO via Synthesis of Cytochrome *c* Oxidase 2 (SCO2) [44], we hypothesized that the decrease in Pten expression in cerebellum and hippocampus from HET-CRE mice could result in an altered expression and/or transcriptional activity of p53 leading to lower SCO2 protein expression and CCO activity.

*Pten* haplo-insufficiency resulted in decreased p53 protein expression in cerebellum of HET-CRE mice (42% of HET, *p*<0.05; **Table 3**, **Figure S3**), accompanied by a lower protein expression of p21 and SCO2, two downstream effectors of p53 (p21 = 54%; *p*<0.005; SCO2 = 45% of HET; *p*<0.05; **Table 3**, **Figure S3**). Although p53 protein level was not found to be significantly different in hippocampus of HET-CRE mice (83% of HET, **Table 3**, **Figure S4**), p21 and SCO2 protein levels were decreased (p21 = 61% of HET; *p*<0.005; SCO2 = 57% of HET; *p*<0.05; **Table 3**, **Figure S4**), suggesting an involvement of p53. The apparent discrepancy between lack of p53 protein changes and decreases in p21 and/or SCO2 can be bridged considering that relatively small changes in p53 expression (or in its post-translational modifications) are amplified through a signaling cascade resulting in more evident changes in p53-downstream targets [45]–[49].

No changes in p53, SCO2 or p21 protein levels were observed at any age in cortex, or at 4–6 and 8–13 weeks in cerebellum (**Figure S3**) or hippocampus (**Figure S4**).

Lower MW (<30 kDa) fragments resulting from caspase-dependent cleavage of p53 during apoptosis [50] were not evidenced by western blots performed with antibodies directed towards either the *N*- (1C12 antibody, Cell Signaling) or *C*-terminus (Invitrogen cat n. 134100) of p53 in all brain regions from HET or HET-CRE mice (not shown) suggesting a limited role for apoptosis in *Pten* haplo-insufficiency.

The differences between hippocampus and cerebellum could be attributed to the tissue-dependent expression of Pten as well as that of p53, p63, and p73, explaining why different brain regions (or human cell types) respond differently to identical cellular damages [51]. In addition, protein expression of mitochondrial Complexes in brains from individuals with autism has been found affected both in a tissue-specific and an age-specific manner [52] and different functional abnormalities in frontal cortex, amygdala-hippocampal (limbic) regions and cerebellum had been reported [53]–[55]. These results indicated that *Pten* haplo-insufficiency in HET-CRE cerebellum and hippocampus resulted in changes in the expression of downstream targets of p53, and ultimately, in changes in CCO activity.

### *Pten* inhibition, p53 levels and cytochrome c oxidase activity in striatal neurons

**Figure 3:**
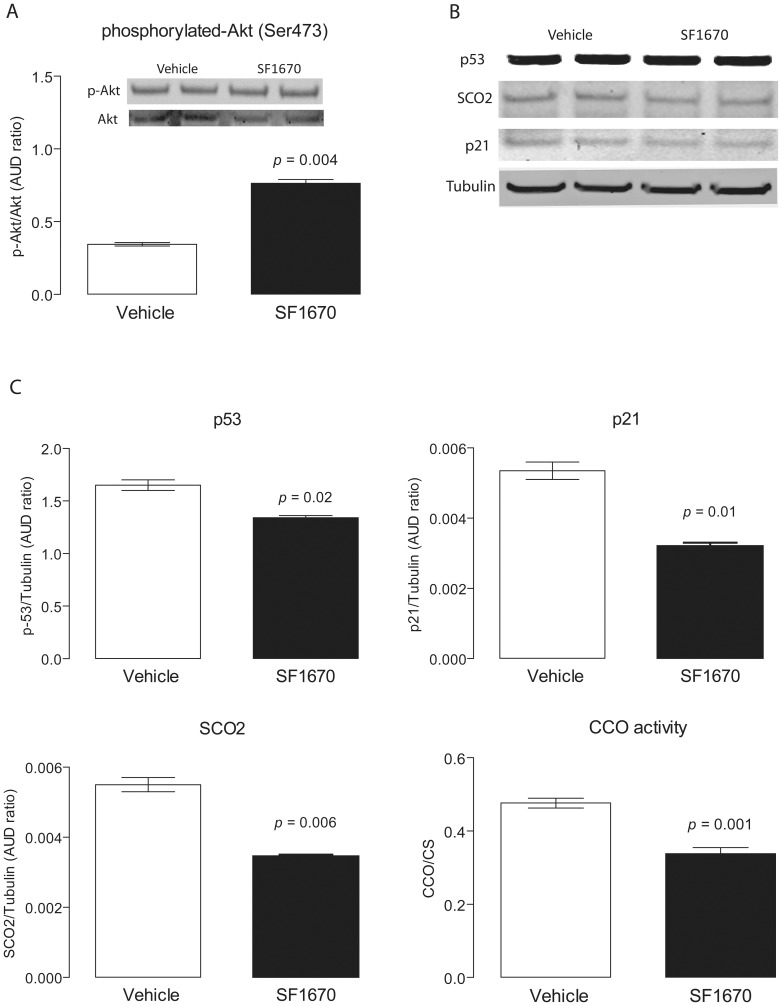
Protein expression of p53, p21 and SCO2 and CCO activity in Pten-deficient striatal neurons. **A.** Representative Western blot and densitometry of p-Akt and total Akt protein levels in striatal neurons. Results are expressed as the ratio between p-Akt and total Akt and reported as mean ± SEM. **B.** Representative Western blots of p53, p21 SCO2 and Tubulin. Forty µg of protein were loaded in each lane. Details on the antibodies used are described in the Materials and Methods. **C** Densitometry results for p53, p21, SCO2 protein expression, normalized by Tubulin (loading control). CCO activity was expressed as nmol×(min×10^6^ cells)^−1^ and reported normalized by citrate synthase. Results are mean ± SEM of experiments in triplicates.

To confirm the results obtained with the *Pten* haplo-insufficient mice, we used a different but still relevant biological model, striatal neurons (STHdh^Q7/Q7^), in which Pten activity had been inhibited by SF1670 [56]. Given that the phosphorylation of Akt at Ser-473 is regulated by PI3K through Pten [57], Western blots against pAkt were performed to evaluate the SF1670-mediated inhibition of Pten (**Figure 3A**). The phosphorylation of Akt (normalized to total Akt) was 2.2-fold higher in SF1670-treated cells (*p* = 0.004), confirming the inhibition of Pten. P53, p21 and SCO2 protein levels in SF1670-treated cells were decreased by 20%, 40% and 38%, respectively, of vehicle-treated neurons (*p* = 0.02; *p* = 0.01; *p* = 0.006; **Figure 3B, C**). In agreement with the results obtained with the *Pten* haplo-insufficient mice, CCO activity was also significantly lower in Pten-deficient neurons than controls (70% of untreated, *p* = 0.003; **Figure 3C**).

### P53 acts as a negative regulator of Pten in HCT 116 cells

To test the hypothesis that direct changes in p53 could modulate Pten activity, we tested the same outcomes as described before but using *p53* ^+/+^, *p53* ^+/−^ and *p53* ^−/−^ human colon cancer cells (HCT 116; **Figure S5**). *P53* gene expression in *p53^+/−^* and *p53^−/−^* cells was 35% and <10% compared to wild type (not shown) and consistent with the p53 protein level of 40% (*p*<0.01) and <2% of *p53* ^+/+^, respectively (**Figures S5A & S5B**). The mtDNA copy number in *p53^+/−^* cells was 1.3-fold of WT (**Figure S5C**) with no apparent mtDNA deletions (consistent with the results obtained with HET-CRE cerebellum aged 8–13 weeks), or changes in MnSOD protein levels (**Figures S5A & S5B**). MtDNA deletions were found significantly increased (2-fold, *p*<0.05; **Figure S5C**) in *p53^−/−^* cells accompanied by a 1.8-fold increase in MnSOD levels (*p*<0.05) relative to WT. To elucidate the link between p53 and mtDNA copy number maintenance [58], the gene expression of the mitochondrial transcription factor A (TFAM) was evaluated in HCT 116 cells. TFAM has been claimed to be required for maintenance of normal levels of mtDNA and coordinating the assembly of multiple DNA molecules into nucleoid-like structures [35], [59]–[61]. The relative TFAM mRNA expression was 3- and 4-fold of controls for *p53* ^+/−^ and *p53* ^−/−^ (*p* = 0.05 vs. controls), respectively, proportional to the p53 gene dosage (**Figure S5C**). These results suggested that p53 (directly or indirectly) affected TFAM gene expression reciprocally, and that this factor (and may be others; [62]) may ultimately control the mtDNA copy number.

The transcript levels of SCO2 were 40% in *p53^+/−^* and 50% in *p53^−/−^* of wild-type cells. In agreement with these results, protein levels of p21 and SCO2 were 63% (*p*<0.001; **Figures S5A & S5B**) and 75% (*p*<0.05; **Figures S5A & S5B**) of *p53^+/+^* in *p53^+/−^* cells. CCO activity was 61% (*p*<0.01; **Figure S5C**) and 21% (*p*<0.01; **Figure S5C**) of WT in *p53^+/−^* and *p53^−/−^* cells, not necessarily accompanied by lower protein expression of CCO subunits (mtDNA-encoded subunits II and III; not shown). Consistent with these results, fibroblasts from patients with SCO2 mutations have decreased CCO activity not associated necessarily with changes in the expression of CCO protein subunits [63].

These results confirmed previous reports that the sole *p53* haplo-insufficiency influences the levels of SCO2 and that of CCO activity [44] but expanded them by including the observation that Pten protein levels are also modulated by p53 (*p53^+/−^* cells had 75% of WT; *p*<0.05; **Figures S5A & S5B**).

Of note, although the decreases in p53 observed in HET-CRE cerebellum and HCT 116 *p53^+/−^* were comparable (40 to 42%; **Table 3** **, Figure S3 and Figure S5**), some of the outcomes in HET-CRE cerebellum (namely, SCO2, CCO/CS and MnSOD) were similar to those observed in *p53^−/−^*, while others fell between those of *p53^−/−^* and *p53^+/−^* (CCO), suggesting synergism between Pten and p53 (negative feedback loop).

## Discussion

Considering that disturbances in energy metabolism underlie some neurological conditions such as schizophrenia, affective disorders, fragile X-associated tremor and ataxia syndrome (FXTAS) and autism [32], [64]–[66], we tested if *Pten* haplo-insufficiency in neural tissues resulted in the occurrence of aberrant social and repetitive behavior and/or mitochondrial dysfunction, and evaluated the mechanism for such dysfunction. To this end, we developed a *Pten* haplo-insufficient model, similar to the one described by Parada's group [14], but based on a two-step breeding process (to reduce the genetic variability between littermates in the second-generation mice). This approach allowed us to generate a mouse model more representative of the heterozygous germline mutations reported in children with autism [9]–[11], [19], [67] while minimizing the risk for cancer development. Our results were indicative of a mechanism that entailed a sustained activation of PI3K/Akt pathway, for which Pten is a negative modulator, followed by a negative feedback loop between Pten and p53 resulting in a specific decrease in CCO activity, ensuing in energy stress in cerebellum and hippocampus.

Symptomatic mice presented lower CCO activity in both cerebellum and hippocampus, deficits that paralleled those in p21 and SCO2 protein expression. It is interesting to note the interplay between Pten, p53 and CCO, given that p53 is the only transcription factor that has been found to be directly linked to CCO activity [44], [68]. Lower SCO2 (a metallochaperone involved in the synthesis of subunit II of CCO and CCO maturation [69]) resulted in impaired CCO activity, along with the occurrence of behavioral abnormalities in 20–29 weeks old mice. It is of interest to note that SCO2 deficiency and/or CCO activity defects have been reported in a number of patients with learning disabilities as well as autism [32], [70]–[73] and that a number of mutated proteins in ASD had been found associated with p53-signaling pathway [13].

**Figure 4:**
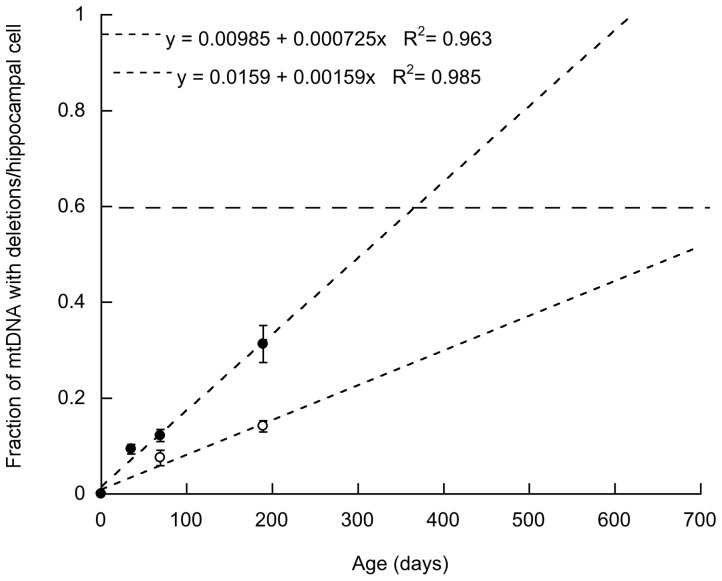
mtDNA deletions in HET-CRE and HET hippocampus accumulate with age. Accumulation of mtDNA with deletions (average of deletions at the segments encoded for CYTB, COX3 and ND4) with age per single hippocampal cell from HET (white circles) and HET-CRE (black circles). The mtDNA deletions (represented as the mean ± SEM) values were fitted to a linear regression (equations shown in the figure) and the goodness of the regression was expressed as r^2^. From the slope of each equation, the copy-error probability was estimated by using the following formulae N_del_ = (N_mtDNA_*P_del_*t * ln 2)/*t*_1/2_, where N_del_ is the number of mtDNA with deletions, t is the age of mice in days throughout their lifetime (estimated as 2 years), N_mtDNA_ = 1,200 mtDNA copy number/cell (experimentally determined in hippocampus by evaluating the ratio of mitochondrial ND1 gene copy number to the nuclear PK gene copy number), and a mtDNA half-life (*t*_1/2_) of 10 d [121]. For HET mice, a low copy-error probability was obtained (9.5×10^−6^) associated with a low incidence of accumulation of deletions with age, becoming more significant towards the end of their life. Higher copy-error probabilities, as that calculated with HET-CRE mice (1.9×10^−5^) lead to a greater accumulation of mtDNA with deletions throughout a simulated mouse life. A biochemical defect on CCO activity is evidenced when the level of a mtDNA deletion exceeds a critical threshold level of 50%–60% [77] (indicated with a dotted line).

Considering that mtDNA deletions were significant in *Pten* haplo-insufficient mice (20 to 29 weeks old) and that p53 has been implicated in the maintenance of mtDNA (this study and others [35], [58], [62]), among other genes [62], it could be argued that clonal expansion of mtDNA with deletions can accumulate over time and outnumber wild-type mtDNA [74]. It has been suggested that the replicative advantage of large-scale deletions is due to a faster completion of replication of smaller mtDNA molecules [75], [76]. Although we observed that mtDNA deletions accumulate in hippocampus with age in HET and, at a 2-fold increase in HET-CRE mice (**Figure 4**) suggesting a higher copy-error probability, to evidence a biochemical defect on CCO activity, the level of a mtDNA deletion needs to exceed a critical threshold level of 50% to 60% [77]–[79], level only reached at 1 year of age in HET-CRE and close 2 years of age in HET according to our simulation (see limitations of simulation in **Figure 4**, legend). Thus, deletions acquired in later adult life of HET do not seem to have sufficient time to express a biochemical defect and/or to reach significant levels by random genetic drift alone, whereas in HET-CRE, and if our model were correct, mtDNA deletions might exacerbate the CCO deficiency initiated earlier by the PTEN-p53-SCO2 axis. Furthermore, it is interesting to note that multiple mtDNA deletions have been reported in association with psychiatric and behavioral disturbances with slow progressive course and adult onset [80]–[82] and possibly in the observed delayed phenotype of Huntington's disease (Napoli et al., 2012, submitted manuscript).

**Figure 5:**
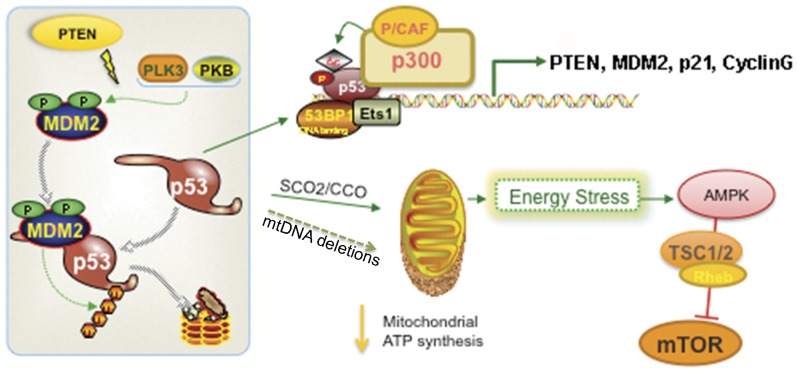
Interplay between Pten and p53 and energy stress response mechanism to mitochondrial dysfunction. Deficits in *Pten* can increase p53 degradation via Akt-mediated phosphorylation of MDM2 or by a feedback loop. Decreased levels of p53 leads to decreased levels of its downstream effectors (p21 and SCO2), subsequent CCO deficiency, leading to increased oxidative stress and energy deficits (this and other reports [44]). This would elicit the energy stress-mediated mTOR activation with consequent brain hypertrophy as observed in our haplo-insufficient *Pten* mice as well as in the *null* mice models. Accumulation of mtDNA deletions (see **Figure 4**) may also ensue in MD contributing to the pathogenesis.

Several lines of evidence connect Pten and p53 functionally (reviewed in [37]), including Akt-mediated phosphorylation of MDM2 allowing MDM2-mediated ubiquitination and degradation of p53 [83]–[85] (**Figure 5**). A feedback loop has also been suggested by the potential of p53 to regulate *Pten* transcription [43]. This loop would explain our results obtained with the HCT 116 model in which the sole p53 haplo-insufficiency results in decreased Pten protein levels. Social deficits present in conditional *Pten null* mice were ameliorated by treatment with rapamycin [15], indicating the involvement of the mTOR (mammalian target of rapamycin complex) pathway. The latter observations, together with our findings, predict a role for an mTOR-mediated response to the earlier energy stress resulting from MD.

Over-expression of Pten in embryonic fibroblasts of transgenic mice showed increased mitochondrial biogenesis and OXPHOS capacity via PGC1α pathway activation [18]. Conversely, decreases in Pten would be expected to result in decreased mitochondria biogenesis and lower MnSOD (PGC1α is a positive regulator of MnSOD transcription [86]). However, our model of Pten haplo-insufficiency seem to point to the activation of a mechanism that does not necessarily involve PGC1α, i.e., tissue-specific regulation of Pten and p53 resulting in lower mitochondrial OXPHOS without significant changes in mitochondrial mass (as judged by CS activity) and increased levels of MnSOD. This apparent discrepancy can be bridged considering the differences in biological models (generalized overexpression of Pten *in utero* vs. specific knock-down of Pten in cerebellum and hippocampus at postnatal stages).

*Pten* haplo-insufficient mice aged 20–29 weeks showed abnormal social and repetitive behavior with some features similar to those present in ASD, *i.e.*, limited preference for social novelty, failure to habituate to a familiar stimulus, social avoidance behavior and repetitive or stereotyped behavior. Of note, atypical social interactions are observed in many psychiatric disorders besides autism, including depression and schizophrenia [87], [88]. In this regard, an argument could be made that *Pten* haplo-insufficient mice would serve as a better model for schizophrenia than autism given that (i) the atypical social behavior observed in this study was not observed until post adolescence when an autistic-like phenotype would require a model in which the behavioral and anatomical changes are present before adolescence (autism requires a diagnosis before 3 years of age [89], [90]) and (ii) mitochondrial dysfunction is present in individuals with schizophrenia [91]. However, (i) schizophrenia is characterized by reduced brain size [92], [93] whereas a subset of individuals with autism presents macrocephaly [9], [94]–[96]; (ii) schizophrenia tends to be associated with reduced function of genes involved in the up-regulation of the PI3K/Akt pathway [97], [98] whereas autism has been associated with loss of function of genes acting as negative regulators of the PI3K/Akt/mTOR pathway [14], [99]; (iii) in this regard, *de novo Pten* mutation had been found strongly associated with increased risk of developing ASD not schizophrenia [100]; and (iv) the difference in the onset and full penetrance of aberrant social behavior in our study vs. those utilizing a *null* model [14], [101] (i.e., our study 8–13 and 20–29 weeks vs. 6–8 and 8–12 weeks for null models [14], [101], respectively) and the phenotypic characteristics (Lhermite-Duclos disease, Cowden syndrome, and ASD, seizures, death vs. aberrant social and repetitive behavior) seem consistent with a *Pten* gene dose effect (i.e., this study, Cre^−/+^; Pten^+/LoxP^ were haploid for Flox-Pten as compared to previous studies, in which Flox-Pten was homozygous) more than a set of developmental processes that result in a post-adolescence behavioral phenotype.

Finally, it is worth mentioning the role of oxidative stress in *Pten* haplo-insufficient mice also observed in CCO-deficient cybrids constructed with mtDNA from patients with sporadic AD [102]. In addition, increased oxidative/nitrative stress might arise from increased production of NO by eNOS, activated by the Akt pathway, which is normally negatively regulated by PTEN; SCO2 deficiency resulting in oxidative DNA damage [103], [104]; and loss/decrease in assembled CCO - as it has been observed with mutations in SCO2 [105] - could also affect the activity of Complex III, the major site for ROS production in mitochondria considering that the respiratory chain can exist as a supercomplex [106]. Although we have not observed changes in SCCR activity in *Pten* haplo-insufficiency, this biochemical assay does not evidence relatively small changes in Complex III activity [107], which could be significant in terms of ROS production.

This study underlines the importance of MD as a contributor to either the development of symptoms and/or severity of the phenotype. The final response to energy stress could be dependent on a combination of genetic (*Pten*, *p53*, other genes) and/or environmental factors [108], [109] increasing the predisposition to abnormal neurodevelopment [110], [111] and resulting in a convergent phenotypic characterized by aberrant social and repetitive behavior. Indeed, energy-requiring processes that rely heavily on mitochondrial OXPHOS and are necessary for typical neurodevelopment, such as neuronal migration in the amygdala-hippocampal region, have been found deficient in individuals with ASD [112]. Alternatively, symptoms attributed to autism as well as a number of neurological disorders [113], [114], may result from an imbalance of excitatory and inhibitory neurotransmission produced by an abnormal GABA catabolism which is linked to mitochondrial function (“GABA shunt”; [115]), possibly compounded by accumulation of mtDNA deletions.

## Materials and Methods

### Loxed *Pten* and Cre-loxed *Pten* mice

Mice expressing the Cre transgene under the control of the neuron-specific enolase (Eno) promoter and a mouse expressing a conditional-ready (i.e., loxP-flanked) phosphatase and tensin homolog (*Pten*) gene were purchased from Jackson laboratories (Tg(EnoCre) mice and *Pten* mice; JAX Stock #5938 and 6440, respectively). After interbreedings of the ensuing 2 generations, a generation of mutant mice in which the *Pten* gene was deleted only in neural tissues (e.g., central nervous system neurons; spinal neurons) was obtained. The benefit of the two-step breeding was a reduction of genetic variability between littermates in the second-generation mice. In addition, genotyping was used to screen for hemizygosity of Cre-recombinase transgene. All other details regarding genotyping of mice and animal housing had been included under **Methods S1**. Detailed information on the mice age, number, gender and test performed for these studies are reported in **Table 1**.

### Ethics Statement

All animal procedures were conducted in strict compliance with the policies on animal welfare of the National Institutes of Health. All experiments performed with animals followed the protocol #16183 (expiration date Dec 2, 2012) approved by IACUC at the University of California Davis.

### Behavioral test procedures

Upon transfer of the mice from the Mouse Biology Program (UC Davis), where they were bred, to the behavioral laboratory facility (UC Davis), mice were first evaluated for general health [30], [116], [117], including body weight, body length, eye, fur and whiskers physical conditions. The animals were housed three to four mice/cage. Mice from the two groups appeared in good general health (checked by a third-party veterinary staff), without any overt impairments, aberrant responses or unusual levels of activity or fighting during the home cage observation periods. Behavioral testing began 4–6 days after arrival into the animal facility. Mice were characterized in assays for sociability and preference for social novelty. Order of testing for the mice was: 1) general health observations upon arrival of mice at the behavioral laboratory facility; 2) social behavior test. Unless otherwise indicated, testing was conducted under fluorescent laboratory lighting. All mice appeared to be healthy at the conclusion of the testing sequence. All details on the behavioral tests had been included in the **Methods S1**.

### Cell Culture conditions and treatment with Pten inhibitor

Conditionally immortalized striatal neuronal progenitor cell lines (STHdh^Q7/Q7^), obtained from the Coriell Cell Repositories, were used in this study [118]. Frozen vials of striatal cells were thawed at 33°C. Cells (4×10^5^) were plated in T75 flasks and grown at 33°C in a humidified atmosphere containing 5% CO_2_ with 20 ml of Dulbecco's Modified Eagle Medium (DMEM) supplemented with 10% FBS (Hyclone #SH30071.03), 10^4^ I.U./ml Penicillin and 10^4^ µg/ml Streptomycin (Gibco), without G418. After 24 h, the media was changed to growth media without G418. When 70% confluent, cells were trypsinized with 3 ml 0.25% Trypsin-EDTA for 5 minutes to dissociate attached cells to the flask and then grown in media with 0.4 mg/ml G418 at 33°C for 2–3 days until 70–80% confluent. For Pten inhibition, cells were grown for 24 hours and then treated with either 3 nM SF1670 (Cellagen Technology, San Diego, CA) [56] or vehicle (DMSO) for another 24 hours at 33°C. At the end of the incubation, cells were washed with PBS, detached by trypsinization and viability was determined as explained above. Average cell viability was 95% regardless of the treatment performed.

Human colorectal carcinoma cell lines (HCT 116; ATCC #CCL-247) whose genotype was *p53^+/+^*, *p53^+/−^*, or *p53^−/−^* were grown in McCoy's 5A modified medium supplemented with 10% fetal bovine serum [119]. The media were supplemented with penicillin 10 U/ml and streptomycin 10 mg/ml. All cell lines were maintained at 37°C under 5% CO_2_ atmosphere. Details on quantitative PCR analysis of HCT 116 cells were given in **Methods S1**.

### Mitochondrial enzymatic and Complex activities

Mitochondria from each brain region was isolated by differential centrifugation and purified through Percoll gradient to obtain non-synaptosomal mitochondria. “Non-synaptosomal mitochondria” includes the mitochondrial fraction minimally contaminated with synaptosomes, i.e., vesicles that arise from nerve terminals during tissue processing which rapidly reseal capturing other non-mitochondrial components [120]. Individual activities of each Complex were tested after lysing the organelles in 20 mM Hepes, pH 7.4 supplemented with proteolytic and phosphatase inhibitors (Sigma, cat # P2714 and P8849). NADH-decylubiquinone oxidoreductase (NQR), NADH-ferricyanide reductase (NFR), Succinate cytochrome *c* reductase (SCCR), Cytochrome *c* oxidase (CCO), ATPase, and citrate synthase activities were evaluated as described before in detail [32].

### Western blotting procedures and analysis

Samples were homogenized in RIPA buffer and the protein concentration was evaluated using a BCA Protein assay kit (Pierce). Proteins were denatured in SDS-PAGE sample buffer (BioRad) plus 5% 2-mercaptoethanol at 100°C for 5 min. Proteins were run on an SDS-PAGE, transferred to PVDF membranes, and probed with mouse monoclonal antibodies reactive to actin (Sigma), β-ATPase (DB Transduction Laboratories), Akt (Cell Signaling), p-Akt (Ser 473, Cell Signaling), MnSOD (Millipore), nitrotyrosine (Millipore), p21 (Santa Cruz), p53 (1C12, Cell Signaling), Pten (Millipore), SCO2 (Proteintech Group), and tubulin (Sigma). Secondary antibodies were all from Invitrogen. Proteins were visualized either with chemiluminescent reagents (ECL) on a Kodak 2000 MM Imager or with fluorescence on an Odyssey imager (LI-COR). The densitometry values were normalized to actin, tubulin or β-ATPase as a loading control.

### Evaluation of mtDNA copy number per cell and mtDNA deletions

Tissues from mice were harvested and total genomic DNA was isolated by using the Puregene kit from Qiagen, following the manufacturer's instructions (See also **Figure S1**). For evaluation of mtDNA copy number per cell, quantitative real-time PCR (QPCR) with dual-labeled probes was performed. The targeted genes were the single-copy nuclear PK and mitochondrial CYTB, ND1, ND4 and COX3. All other details were included in **Methods S1**.

### Statistical Analyses

The experiments were run in duplicate or triplicates and repeated three times in independent experiments unless noted otherwise. Data were expressed as mean ± SEM. The data were evaluated by using the *t*-test (StatSimple v2.0.5; Nidus Technologies, Toronto, Canada). For all comparisons, significance was set at *p*≤0.05.

## Supporting Information

Method S1 **Detailed information on some of the methods utilized in this study.**
(DOCX)

Figure S1 **Genotyping of HET-CRE mice for wild-type and truncated Pten.** Genomic DNA from various tissues from HET (Pten^+/loxP^) and HET-CRE mice (Cre^+/−^; Pten^+/loxP^) was extracted as described in Materials and Methods. Cre-mediated Pten deletion in HET-CRE mice.
(TIF)

Figure S2 **Nitrated β-ATPase levels in 20–29 weeks old mice cerebellum.** Representative Western blots and densitometry results of nitrated β-ATPase in 20–29 weeks old mice cerebella. Nitrotyrosine levels were normalized by total β-ATPase.
(TIF)

Figure S3 **Pten, p53, p21 and SCO2 levels in 4–29 weeks old mice cerebellum.** **A**. Representative Western blots of Pten, p53, p21, SCO2 and respective actin levels in 8–13 and 20–29 weeks old mice cerebella (20–30 µg protein per lane). **B**. Average expression of p53, p21 and SCO2 in 4–6, 8–13 and 20–29 weeks old mice cerebella. HET: white bars; HET-CRE: black bars. Statistically significant *p* values (*p*<0.05) calculated using Student's *t* test are also shown.
(TIF)

Figure S4 **Pten, p53, and downstream effectors levels in 4–29 weeks old mice hippocampus.** **A**. Representative Western blots of Pten, p53, p21 and SCO2 (and respective actin) levels in 8–13 and 20–29 weeks old mice hippocampus. Twenty to thirty µg of proteins were loaded per lane. **B**. Average expression of p53, p21 and SCO2 in 4–6, 8–13 and 20–29 weeks old mice. HET: white bars; HET-CRE: black bars. Statistically significant p values (p<0.05) calculated using Student's t test are also shown.
(TIF)

Figure S5 **Protein expression of p53 downstream effectors and CCO activity in** ***p53*** **haplo-insufficient HCT 116 cells.** Representative Western blots (**A**) and densitometry (**B**) of p53, p21, SCO2, Pten, MnSOD and actin (loading control) in controls and *p53* haplo-insufficient HCT 116 cells. Thirty µg of protein were loaded into each lane if a 4–15% gels. MnSOD expression was normalized by the beta subunit of ATPase (β-ATPase, mitochondrial loading control). Results are expressed as units of densitometry (AUD) normalized by actin, and reported as mean ± SEM (% of controls) of at least two experiments in quadruplicate. Significant with * *p*<0.05; **p<0.01; ***p<0.001 to *p53^+/+^*. MtDNA copy number, mtDNA deletions, CCO activity and TFAM mRNA levels (**C**) in *p53* haplo-insufficient HCT 116 cells (*p53^+/−^* and *p53^−/−^*) are also shown as percentage of controls cells (*p53^+/+^*). Percentage of mtDNA deletions were calculated as follows: 100−(100×mtDNA gene ratio *p53^+/−^* : *p53^+/+^*) or 100−(100×mtDNA gene ratio *p53^−/−^* : *p53^+/+^*). CCO activity is expressed as nmol/min/mg protein and reported normalized by citrate synthase (CS) as % of controls. Results are mean ± SEM. Significant with *p<0.05; **p<0.01; ***p<0.001 to *p53^+/+^*.
(TIF)
