# Supplementary material for: Mitochondrial Dysfunction in Pten Haplo-Insufficient Mice with Social Deficits and Repetitive Behavior: Interplay between Pten and p53
Source: PLoS One. 2012 Aug 10;7(8):e42504. doi: 10.1371/journal.pone.0042504 (PMC3416855; doi:10.1371/journal.pone.0042504)
Supplement: Method S1 — Detailed information on some of the methods utilized in this study. (DOCX) [file pone.0042504.s001.docx]

**METHODS S1**

***Chemicals and biochemicals***

EDTA, EGTA, sodium succinate, mannitol, sucrose, and HEPES were all purchased from Sigma (St. Louis, MO). Tris-HCl, glycine, NaCl and KCl were purchased from Fisher (Pittsburg, PA). Bovine serum albumin (fatty-acid free) was obtained from MP Biomedicals. All reagents were of analytical grade.

***Loxed Pten and Cre-loxed Pten mice:*** Mice expressing the CRE transgene under the control of the neuron-specific enolase (Eno) promoter and a mouse expressing a conditional-ready (i.e.,loxP-flanked) phosphatase and tensin homolog (*Pten*) gene were purchased from Jackson laboratories (Tg(EnoCre)) mice and *Pten* mice (JAX Stock #5938 and 6440, respectively) and bred at the Mouse Biology Facility at University of California at Davis. After interbreedings of the ensuing 2 generations, a generation of mutant mice in which the *Pten* gene was deleted only in neural tissues (e.g., central nervous system neurons; spinal neurons) was obtained. The production of the conditionally mutated *Pten* mice required for study required 2 breeding steps. The first breeding Nse-Cre mice were bred with *Pten*^+/loxP^ mice. The resulting Cre^+/-^*Pten*^+/LoxP^ mice were then bred to attempt to produce 19% of the pups Cre-homozygous *Pten*^LoxP/LoxP^. No homozygous *Pten*^loxP/loxP^ mice carrying the Cre transgene after 9 intermatings between the Cre-*Pten* (Cre^+/-^;*Pten*^+/loxP^) heterozygous mice were obtained because this manipulation appeared to have resulted *intra*-utero lethality, or cannibalism by the mother. Additionally, three separate crosses (parallel or sequential intermatings) between a homozygous *Pten*/*Pten* floxed and Cre-*Pten* (Cre^+/-^; *Pten*^+/+^) heterozygous mice did not result in any wild type *Pten* homozygous progeny carrying a Cre transgene. The benefit of the two-step breeding scheme was to obtain a reduction of genetic variability between littermates in the second-generation mice. The conditional mice were then transferred from the Mouse Biology Facility to the Mouse Behavioral Facility on Campus where they were checked for their general health before performing the behavioral tests.

All procedures were conducted in strict compliance with the policies on animal welfare of the National Institutes of Health and the University of California at Davis (stated in the “Guide for the Care and Use of Laboratory Animals,” Institute of Laboratory Animal Resources, National Research Council, 1996 edition), and approved by the University of California at Davis Animal Care and Use Committee.

***Genetic background***: Nse-Cre recombinase construct was microinjected into (C57BL6 x SJL) F1 embryos and the founder mouse line was cross into a C57BL/6 mouse line. The 129S4/SvJae derived LW-1 embryonic stem (ES) cells harboring the *Pten*^LoxP/+^ were injected into BALB/c blastocysts and the chimeric mice were crossed with BALB/cAnNTac mice. Ultimately, these mice were backcrossed into a C57BL6/6 background at least five generations.

***Genotyping mice:*** Five different tissue types (hippocampus, cortex, cerebellum, liver and heart), were extracted through Gentra Puregene Tissue Kit from Qiagen (cat no. 158622). DNA concentrations were determined through Tecan Microplate Reader and all samples were normalized to 50 ng/µl. DNA was extracted as described previously [[1](#_ENREF_1)]. Two sets of Cre genotyping primers were used: forward primer 1, 5’-GAT CTC CGG TAT TGA AAC TCC AGC-3’, forward primer 2,5’-CAA ATG TTG CTT GTC TGG TG – 3’, the reverse primer 1, 5’- GCT AAA CAT GCT TCA TCG TCG G-3’ and reverse primer 2, 5’- GTC AGT CGA GTG CAC AGT TT-3’. PCR amplification was done using the Taq DNA polymerase consisting of 2.5 μl of 10x Buffer, 1.7 μl of 25 mM MgCl_2_, 0.5 μl of 10mM dNTP mix, 0.5 μl of each primers from set 1, 0.2 μl of each primers from set 2, 1 μl of sample with 50 ng total of DNA, 0.2 μl of Taq enzyme and 17.7 μl of MilliQ water for the total reaction volume of 25 μl. The following cycling conditions were used: 94°C for 3 min, 10 cycles of 94°C for 15 s 65°C for 30 s (with 1°C decrease at each cycle), and 72°C for 40 s, following with 30 cycles of 94°C for 15 s, 55°C for 30 s, and 72°C for 40 s. Final elongation was done for 5 min at 72°C with a 15°C forever hold step. The PCR products were run on agarose gels, and the expected sizes were 2000 bp for wild-type and 2000 and 650 bp for Cre. Genotyping was used to screen for hemizygosity of Cre-recombinase transgene in all mice. *Pten* genotyping primers were obtained from Lesche et al. [[2](#_ENREF_2)] with one forward primer P1, 5-ACTCAAGGCAGGGATGAGC-3, and two reverse, P2 5-AATCTAGGGCCTCTTGTG CC-3, P3 5-GCTTGATATCGAATTCCTGCAGC-3. PCR amplification was done using the Qiagen Taq DNA polymerase consisting of 2.5 μl of 10x Buffer, 1.7 μl of 25 mM MgCl_2_, 0.5 μl of 10 mM dNTP mix, 0.5 μl of each of the three primers, 1 μl of sample with 50 ng total of DNA, 0.2 μl of Taq enzyme and 17.6 μl of MilliQ water for the total reaction volume of 25 μl. The following cycling conditions were used: 94°C for 3 min, 10 cycles of 94°C for 15 s 65°C for 30 s (with 1°C decrease at each cycle), and 72°C for 40 s, following with 30 cycles of 94°C for 15 s, 55°C for 30 s, and 72°C for 40 s. Final elongation was done for 5 min at 72°C with a 15°C forever hold step. The efficiency of the Cre-mediated deletion of *Pten* was deduced from the ratio of the mutant truncated *Pten* to the wild-type *Pten* in various adult mouse tissues (10-12 weeks old) from HET-CRE mice (Cre^+/-^; Pten^+/loxP^). For the particular HET-CRE mouse samples run on the gel shown under Figure S1, (mouse #88) Cre-mediated *Pten* deletion was less than 20% in liver and heart, but it was significant in neural tissues (76%, 60%, and 62% *Pten* deletion in hippocampus, cerebellum, and brain cortex, respectively).

***Animal health, housing conditions and behavior***

***General health:*** Upon transferring the mice from the breeding Facility on Campus to the Mouse Behavioral, mice were first evaluated for general health [[3-5](#_ENREF_3)], including body weight, eye, fur and whiskers physical conditions. Records were taken for body weight and length. The animals were housed three to four mice per cage. Behavioral testing began 4-6 days after arrival into the animal facility. Mice from the two groups appeared in good general health, without any overt impairments, aberrant responses or unusual levels of activity or fighting during the home cage observation periods.

***Animal housing:*** All animals were housed with three to four mice *per* plastic tub cage, and provided with Purina 5058 chow and water *ad libitum*. The housing room was maintained at 23°C on a 12-h light/dark cycle (lights off at 6 pm).

***Behavioral tests: Sociability and preference for social novelty.*** A mouse social approach task was used to assess sociability, the tendency to spend time with another conspecific, and preference for social novelty, the ability to discriminate and choose between familiar and new conspecifics [[5](#_ENREF_5),[6](#_ENREF_6)]. The apparatus used was a rectangular, three-chambered box fabricated from clear polycarbonate. Dividing walls had retractable doorways allowing access into each chamber. The Topscan tracking system was used to track animals in real time and outputs the data in terms of the animal location and sniffing of the test animal to the cages. The chambers of the apparatus were cleaned with water and dried with paper towels between each trial. At the end of each test day, the apparatus was sprayed with 70% ethanol and wiped clean with paper towels.

***Procedure:*** A) Habituation. The test mouse was first placed in the middle chamber and allowed to explore for ten min, with the doorways into the two side chambers open. Each of the two sides contained an empty wire cage (Galaxy Cup, Spectrum Diversified Designs, Inc., Streetsboro, Ohio). The wire cages were 11 cm in height, with a bottom diameter of 10.5 cm and bars spaced 1 cm apart. A weighted cup was placed on the top of each cage to prevent climbing by the test mice. Each wire cage was used only once per day, and all cages were washed with soap and water at the end of each test day. B) Sociability. After the habituation period, the test mouse was enclosed in the center compartment of the social test box, and an unfamiliar mouse (stranger 1; a C57BL/6J male), further described below, was enclosed in one of the wire cages and placed in one of the side chambers. The location for stranger 1 alternated between the left and right sides of the social test box across subjects. Following placement of stranger 1, the doors were re-opened, and the subject was allowed to explore the entire social test box for a ten-minute session. Measures were taken of the amount of time spent in each chamber and the number of entries into each chamber by the automated testing system (Topscan software). In addition, this software also scored time spent sniffing each wire cage. Stranger mice were adult male C57BL/6J (JAX), and were housed in cages separate from and distant to the cages housing the subject mice, to avoid visual, auditory, and olfactory contact. Strangers had no previous physical contact with the subjects, and were kept in a separate location from the subjects on the day of testing. Several days before the start of social testing, the mice serving as strangers were habituated to the wire cages in the social apparatus for 5–10 min per day, for at least five days. Each stranger was used only once per day, and the strangers for the sociability test and the social novelty tests were taken from separate cages. Containing the stranger mouse in a wire cage served the purpose of preventing aggressive and sexual interactions, as well as ensuring that all social approaches were initiated only by the subject mouse. The empty wire cage served as a control for the properties of the container, in addition to serving as inanimate object with no social valence. C) Preference for social novelty. At the end of the ten-minute sociability test, each mouse was further tested in a third ten-minute session to quantitate preference to spend time with a new stranger. A new unfamiliar mouse was placed in the wire cage that had been empty during the previous ten-minute session. The test mouse had a choice between the first, already-investigated, now-familiar mouse (stranger 1) and the novel unfamiliar mouse (stranger 2). As described above, measures were taken of the amount of time spent in each chamber, the number of transitions between chambers of the apparatus, and time spent sniffing each wire cage. **Statistical analysis:** Each mouse was tested separately in the behavioral assays. Data from the two components of the social behavior test (sociability and social novelty) were analyzed using two-tailed Student t-test. For all comparisons, significance was set at *p* ≤ 0.05; however, outcomes were considered relevant and worth reporting when the power of the test was ≥ 0.900.

***Self-grooming.*** Ten HET and six HET-CRE mice (see **Table 1**) aged 20-29 weeks were scored for spontaneous grooming behavior as described by others [[7](#_ENREF_7)]. Each mouse was placed individually into a standard mouse cage at 20-22ºC with < 1-cm layer of bedding. This bedding was included to reduce neophobia and it was thin enough to prevent digging, a potentially competing behavior. After a 10-min habituation period in the test cage, each mouse was scored with a stopwatch for 15- min for cumulative time spent grooming any body part. Self-grooming was analyzed with ANOVA.

***Evaluation of mtDNA copy number per cell and mtDNA deletions:*** Genomic DNA was first isolated from 1 x 10^6^ HTC-116 cells using the Gentra Puregene Cell and Tissue Kit (Qiagen #158388) following the manufacturer’s recommendations for cultured cells. Concentration and purity of DNA was measured at an absorbance of 260 nm and 280 nm using the Tecan i-control 1.6 software (v. 1.6.19.2) on a Tecan infinite M200 Nanoquant (Tecan, Austria, GmbH). Following DNA extraction, changes in mtDNA copy number were evaluated by dual labeled qPCR. The gene copy number of cytochrome *b*, ND1 and ND4 were normalized by a single copy nuclear gene (pyruvate kinase). Because minor changes in the efficiency of the amplification of each gene can result in large changes in the ratio of mtDNA per cell, the mitochondrial primers were designed for regions of low polymorphisms (<1%). Species-specific primers were selected using the Primer Express 3 software (Applied Biosystems). Human primers for PK were: forward 5’-AGCCCAAATGGCCTTGAAG-3’; reverse 5’-AGAGACAGAATGCCAGTGAGCTT-3’; primers for CYTB were: forward 5’-CACGATTCTTTACCTTTCACTTCATC-3’; reverse 5’-TGATCCCGTTTCGTGCAAG-3’. The probes used were from Roche UPL library, Locked Nucleic Acids, short hydrolysis probes, labeled at the 5’ end with fluorescein and at the 3’ end with a dark quencher dye (#11 for PK, cat no:04685105001; #10 for CYTB, cat no: 04685091001). ND1 primers and probe were: forward 3485-3504, 5’-CCCTAAAACCCGCCACATCT-3’; reverse 3532-3553, 5’-CCCTAAAACCCGCCACATCT-3’; probe 3506-3529, 5’ FAM-CCATCACCCTCTACATCACCGCCC-BHQ-3’. ND4 primers and probe were the following: forward 12087-12109, 5’-CCATTCTCCTCCTATCCCTCAAC-3’, reverse 12140-12170, 5’ CACAATCTGATGTTTTGGTTAAACTATATTT-3’, probe 12111-12138, 5’FAM-CCGACATCATTACCGGTTTTCCTCTTG-BHQ-3’. qPCR was performed on a Mastercycler EP Realplex thermocycler (Eppendorf, Westbury, NY). The corresponding real time PCR efficiencies for each mitochondrial and nuclear gene amplification were calculated according to the equation: E=10^(-1/slope)^ -1 according to the Mastercycler EP manual. After establishing the linear response between Ct number and template amount (25, 12.5, 6.25, 3.13 and 1.56 ng total per reaction), efficiencies for each gene were 101 ± 3% and 102 ± 4% for PK and CYTB, and respectively, 98.6 ± 0.3% % for ND1 and 101 ± 3% for ND4. qPCR was performed using 96-well PCR plates with TaqMan Universal Mastermix (Applied Biosystems) with 400 nM of each primer, 80 nM of fluorogenic Roche Universal Library probe and 5 µl of 3.13 ng total of template DNA per reaction. Amplification was performed using the default cycling parameters of 2 minutes at 50°C, 10 minutes at 95°C, and 40 cycles of 15 seconds at 95°C and 60 seconds at 60°C. Products were run on a gel, purified (QIAquick Gel Extraction Kit, Qiagen) and sequenced to check for primer specificity. The mean cycle time obtained by double derivatives (CalqPlex algorithm; Eppendorf, Westbury, NY) was designated as Ct. The detection parameters were set as follows: threshold calqplex, automatic baseline, and drift correction on. Relative mtDNA copy number per cell was assessed by comparative Ct method, using the following equation: mtDNA/nDNA = 2^-ΔCt^, where ΔCt = Ct_mitochondrial_- Ct_nuclear_. Each sample was analyzed in triplicates. Positive and negative controls were run in each plate. mtDNA deletions were considered if the mean ND4/ND1, CYTB/ND1, and COX3/ND1 ratios were lower than the lowest limit of the 99% CI of control values.

***Real-time quantitative PCR analysis of HCT-116 cells:*** Total RNA was isolated by using TRIzol (Invitrogen). For quantitative real-time PCR, cDNAs were synthesized using Advantage RT-for PCR kit according to the manufacture’s protocol (BD Biosciences), followed by amplification of the target cDNAs with SYBR Green-based real time PCR in Mastercycler realplex system with RealMasterMix (Eppendorf). The mRNA levels were calculated to the corresponding of glyceraldehyde-3-phosphate dehydrogenase (GAPDH) used as housekeeping gene as 2^-(Ct target gene – Ct house keeping gene)^[[8](#_ENREF_8)]. For quantification, 20 ng of cDNA was mixed with a buffer containing 4 mM MgCl_2_, 0.4 mM dNTPs, 0.2 µM of forward and reverse primers, SYBR Green I dye, and 0.25 U HotMaster Tag DNA polymerase made up to a final volume of 20 µl. The PCR reaction was performed as follows: initial denaturing at 95˚C for 2 min and 40 cycles at 95˚C for 15s, 55˚C for 20s, and 68˚C for 30s. A melting curve was systematically analyzed in order to check the specificity of the PCR sample. The primers utilized were for p53 forward 5’-TAACAGTTCCTGCATGGGCCGC-3’ and reverse 5’-AGGACAGGCACAAACACGCACC-3’; for SCO2 forward 5’-GCAGCCTGTCTTCATCACTGTGGACC-3’ and reverse 5’-CCGCACACTGTCTGAGATCTGCTC-3’, for TFAM forward 5'-CCCAGATGCAAAAACTACAGAACTAA-3' and reverse 5'-TCCGCCCCTATAAGCATCTTGA-3'. The efficiencies of these primers were tested with the plasmid that contains each cDNA of target genes in extracts from HCT116 cell lines, resulting in values of 0.875 to 0.995.

REFERENCES

1. Giulivi C, Ross-Inta C, Omanska-Klusek A, Napoli E, Sakaguchi D, et al. (2011) Basal Bioenergetic Abnormalities in Skeletal Muscle from Ryanodine Receptor Malignant Hyperthermia-susceptible R163C Knock-in Mice. J Biol Chem 286: 99-113.

2. Lesche R, Groszer M, Gao J, Wang Y, Messing A, et al. (2002) Cre/loxP-mediated inactivation of the murine Pten tumor suppressor gene. Genesis 32: 148-149.

3. Crawley JN (1999) Behavioral phenotyping of transgenic and knockout mice: Experimental design and evaluation of general health, sensory functions, motor abilities, and specific behavioral tests. Brain Research 835: 18-26.

4. Crawley JN (2000) What's wrong with my mouse? Behavioral phenotyping of transgenic and knockout mice. Second Edition John Wiley & Sons, Inc., New York

5. Moy SS, Nadler JJ, Perez A, Barbaro RP, Johns JM, et al. (2004) Sociability and preference for social novelty in five inbred strains: An approach to assess autistic-like behavior in mice. Genes, Brain and Behavior 3: 287-302.

6. Nadler JJ, Moy SS, Dold G, Trang D, Simmons N, et al. (2004) Automated apparatus for quantitation of social approach behaviors in mice. Genes, Brain and Behavior 3: 303-314.

7. Silverman JL, Tolu SS, Barkan CL, Crawley JN (2010) Repetitive self-grooming behavior in the BTBR mouse model of autism is blocked by the mGluR5 antagonist MPEP. Neuropsychopharmacology : official publication of the American College of Neuropsychopharmacology 35: 976-989.

8. Livak KJ, Schmittgen TD (2001) Analysis of relative gene expression data using real-time quantitative PCR and the 2(-Delta Delta C(T)) Method. Methods 25: 402-408.
