# Supplementary figures and images for: Mitochondrial Dysfunction in Pten Haplo-Insufficient Mice with Social Deficits and Repetitive Behavior: Interplay between Pten and p53
Source: PLoS One. 2012 Aug 10;7(8):e42504. doi: 10.1371/journal.pone.0042504 (PMC3416855; doi:10.1371/journal.pone.0042504)

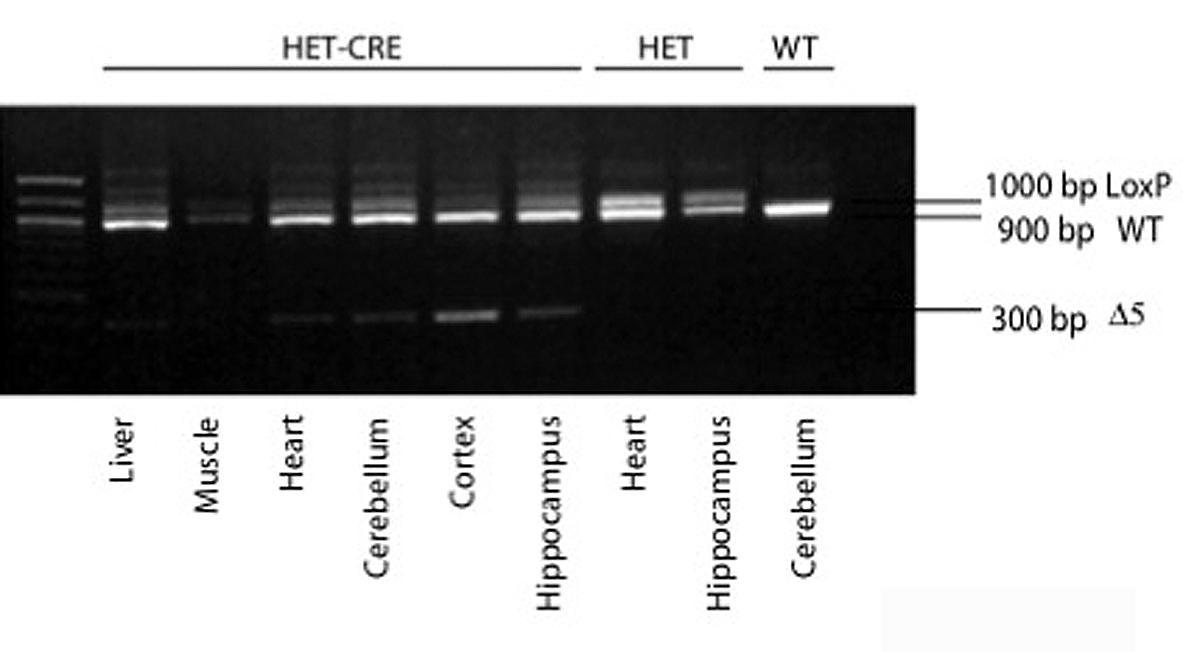

Supplement: Figure S1 — Genotyping of HET-CRE mice for wild-type and truncated Pten. Genomic DNA from various tissues from HET (Pten+/loxP) and HET-CRE mice (Cre+/−; Pten+/loxP) was extracted as described in Materials and Methods. Cre-mediated Pten deletion in HET-CRE mice. (TIF) [file pone.0042504.s002.tif]

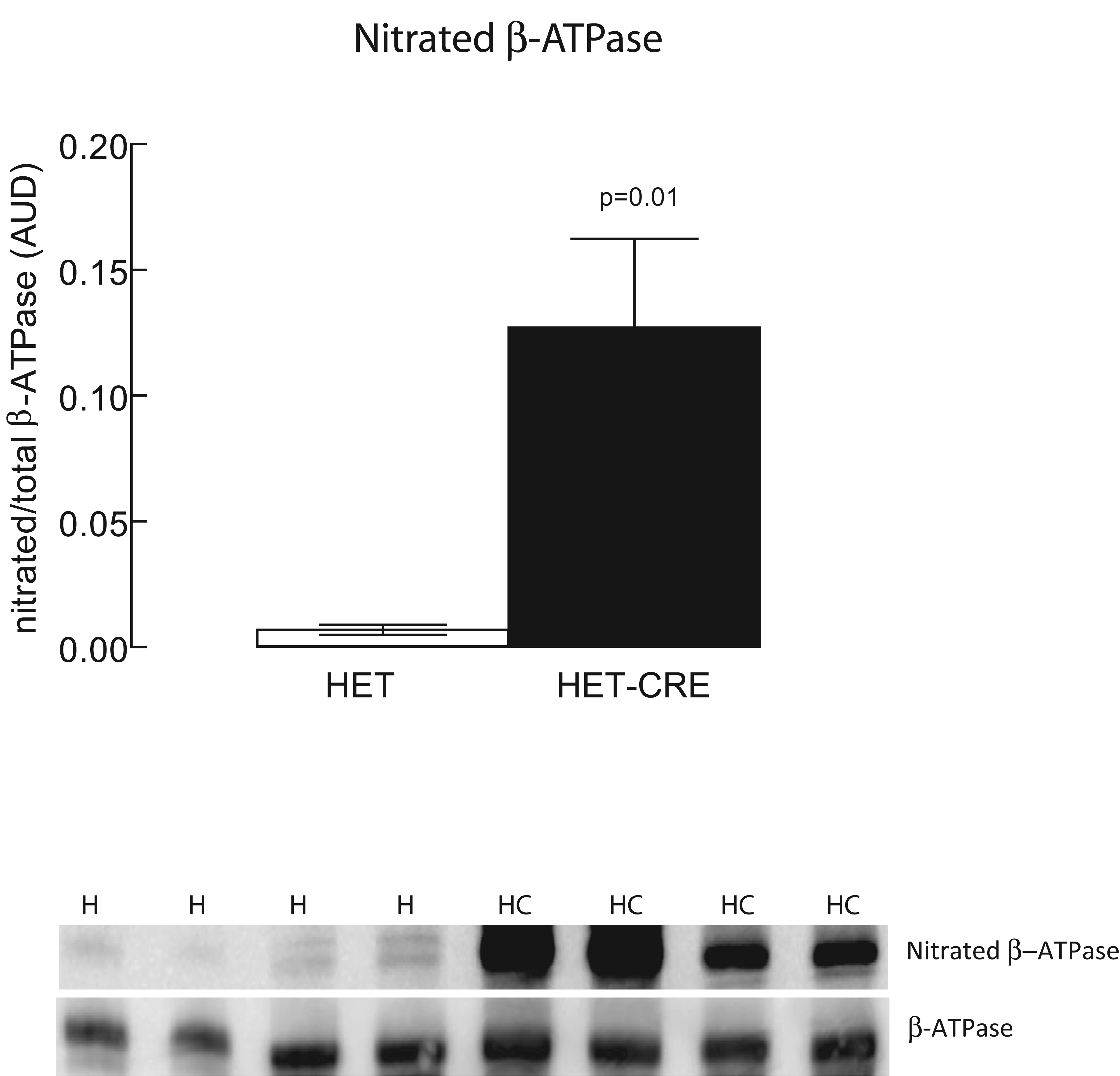

Supplement: Figure S2 — Nitrated β-ATPase levels in 20–29 weeks old mice cerebellum. Representative Western blots and densitometry results of nitrated β-ATPase in 20–29 weeks old mice cerebella. Nitrotyrosine levels were normalized by total β-ATPase. (TIF) [file pone.0042504.s003.tif]

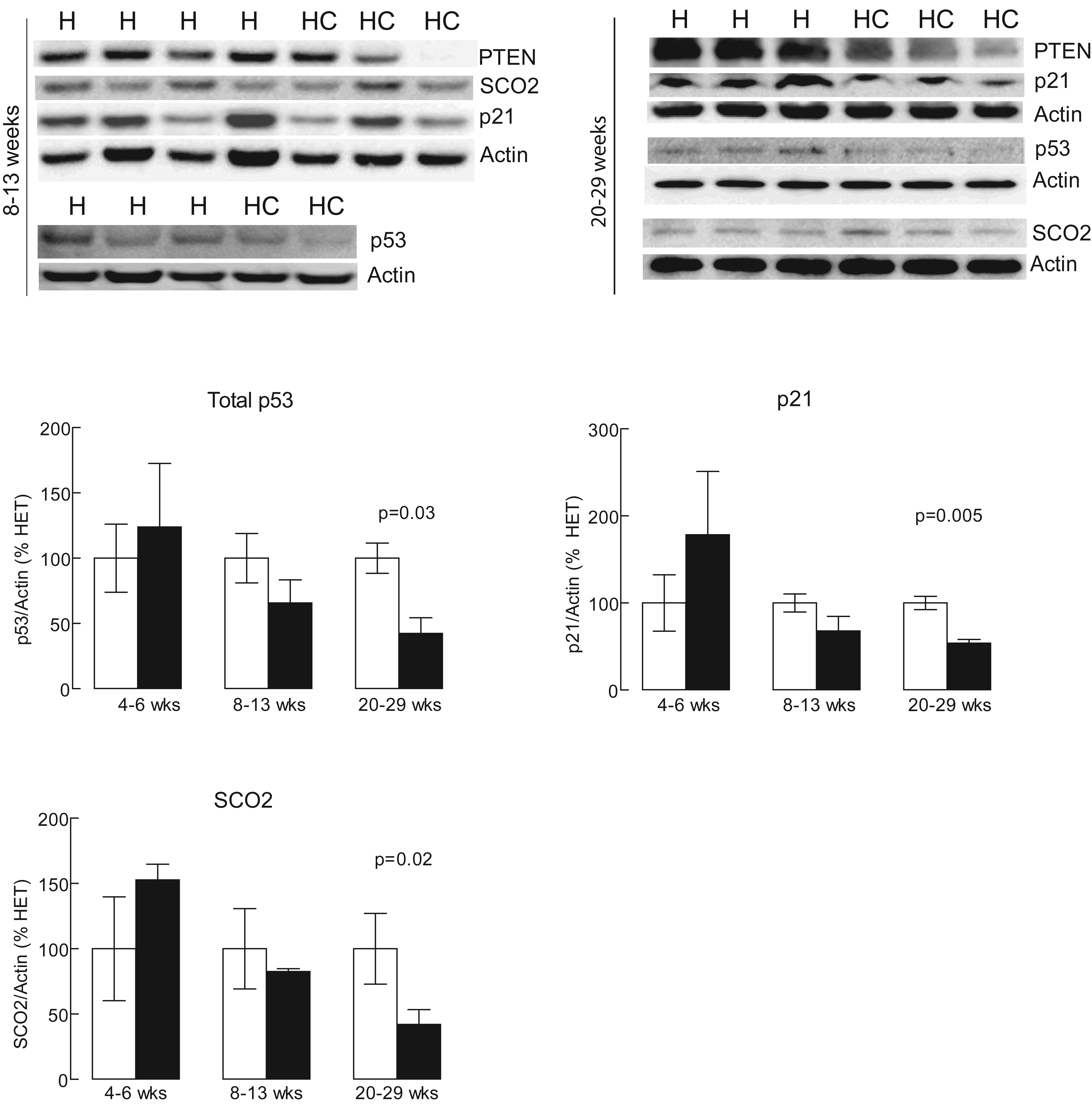

Supplement: Figure S3 — Pten, p53, p21 and SCO2 levels in 4–29 weeks old mice cerebellum. A. Representative Western blots of Pten, p53, p21, SCO2 and respective actin levels in 8–13 and 20–29 weeks old mice cerebella (20–30 µg protein per lane). B. Average expression of p53, p21 and SCO2 in 4–6, 8–13 and 20–29 weeks old mice cerebella. HET: white bars; HET-CRE: black bars. Statistically significant p values (p<0.05) calculated using Student's t test are also shown. (TIF) [file pone.0042504.s004.tif]

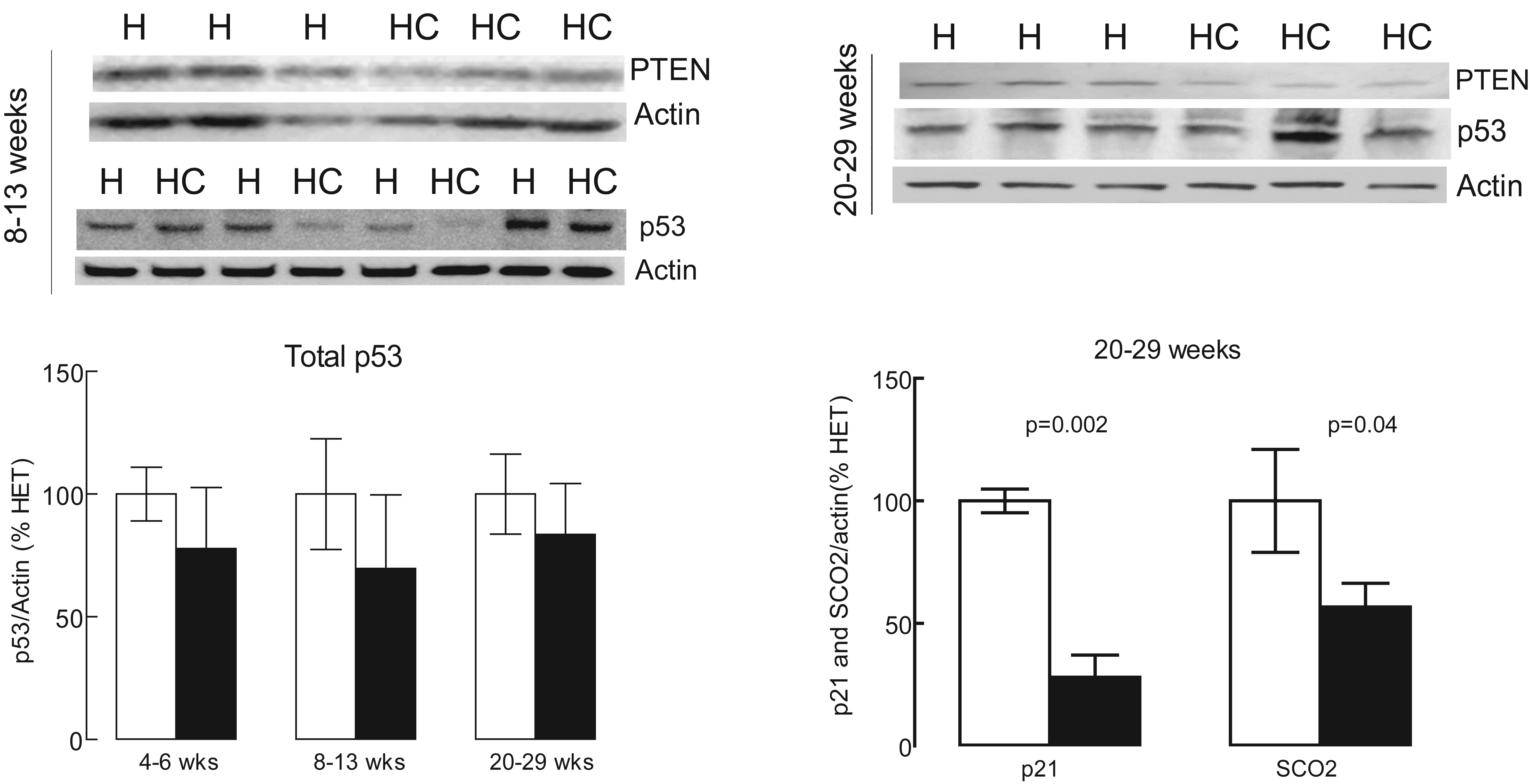

Supplement: Figure S4 — Pten, p53, and downstream effectors levels in 4–29 weeks old mice hippocampus. A. Representative Western blots of Pten, p53, p21 and SCO2 (and respective actin) levels in 8–13 and 20–29 weeks old mice hippocampus. Twenty to thirty µg of proteins were loaded per lane. B. Average expression of p53, p21 and SCO2 in 4–6, 8–13 and 20–29 weeks old mice. HET: white bars; HET-CRE: black bars. Statistically significant p values (p<0.05) calculated using Student's t test are also shown. (TIF) [file pone.0042504.s005.tif]

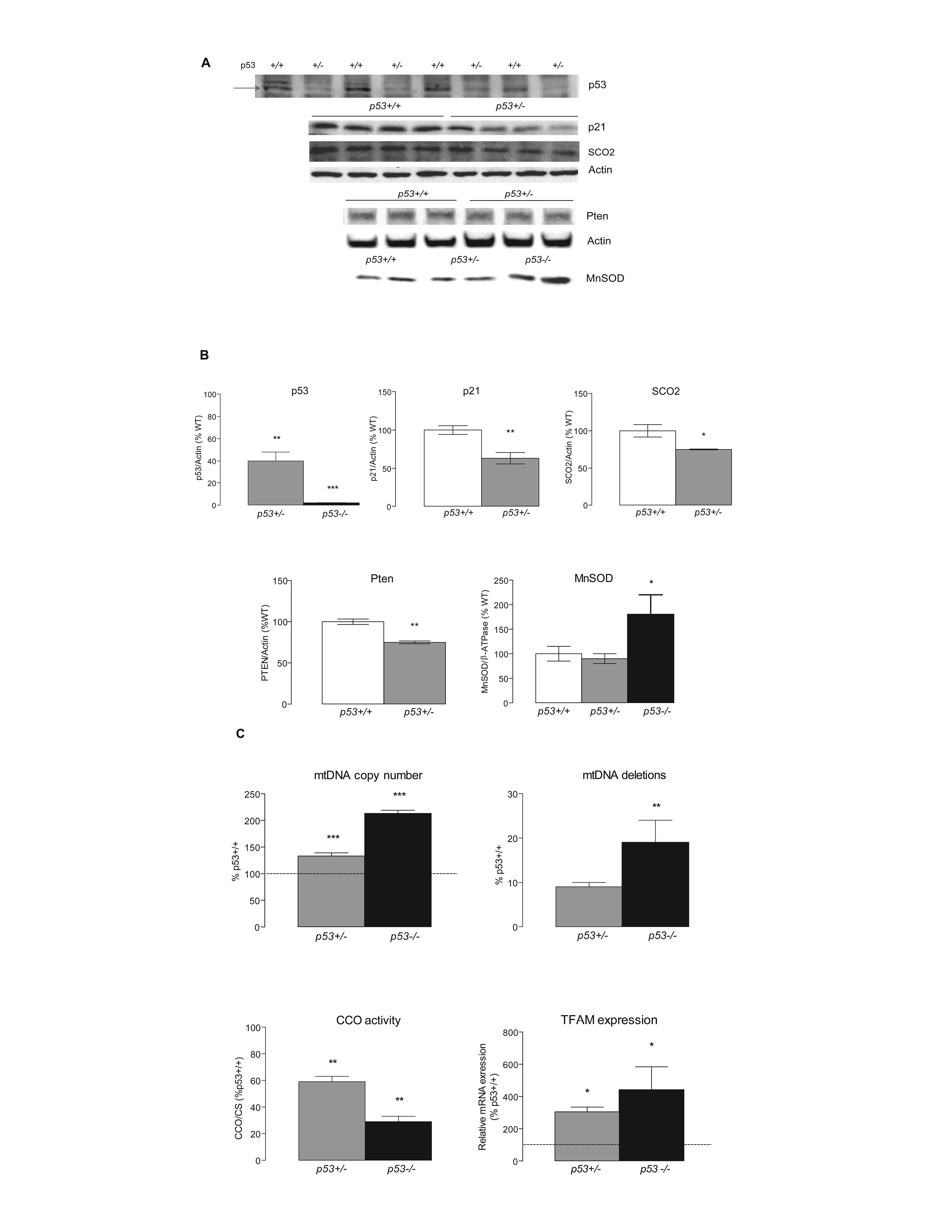

Supplement: Figure S5 — Protein expression of p53 downstream effectors and CCO activity in p53 haplo-insufficient HCT 116 cells. Representative Western blots (A) and densitometry (B) of p53, p21, SCO2, Pten, MnSOD and actin (loading control) in controls and p53 haplo-insufficient HCT 116 cells. Thirty µg of protein were loaded into each lane if a 4–15% gels. MnSOD expression was normalized by the beta subunit of ATPase (β-ATPase, mitochondrial loading control). Results are expressed as units of densitometry (AUD) normalized by actin, and reported as mean ± SEM (% of controls) of at least two experiments in quadruplicate. Significant with * p<0.05; **p<0.01; ***p<0.001 to p53+/+. MtDNA copy number, mtDNA deletions, CCO activity and TFAM mRNA levels (C) in p53 haplo-insufficient HCT 116 cells (p53+/− and p53−/−) are also shown as percentage of controls cells (p53+/+). Percentage of mtDNA deletions were calculated as follows: 100−(100×mtDNA gene ratio p53+/− : p53+/+) or 100−(100×mtDNA gene ratio p53−/− : p53+/+). CCO activity is expressed as nmol/min/mg protein and reported normalized by citrate synthase (CS) as % of controls. Results are mean ± SEM. Significant with *p<0.05; **p<0.01; ***p<0.001 to p53+/+. (TIF) [file pone.0042504.s006.tif]
